# Seroprevalence and Risk Factors of Contagious Bovine Pleuropneumonia in Selected Districts of North Gondar Zone, Ethiopia

**DOI:** 10.3389/fvets.2021.626253

**Published:** 2021-02-26

**Authors:** Wassie Molla, Wudu Temesgen Jemberu, Sefinew Alemu Mekonnen, Getachew Tuli, Gizat Almaw

**Affiliations:** ^1^Department of Veterinary Epidemiology and Public Health, College of Veterinary Medicine and Animals Sciences, University of Gondar, Gondar, Ethiopia; ^2^National Animal Health Diagnostic and Investigation Centre (NAHDIC), Sebeta, Ethiopia

**Keywords:** cattle, contagious bovine pleuropneumonia, *Mycoplasma mycoides* subspecies *mycoides*, North Gondar, risk factor, seroprevalence

## Abstract

Contagious bovine pleuropneumonia (CBPP) is an infectious and highly contagious respiratory disease of cattle and water buffalo, which is caused by the *Mycoplasma mycoides* subspecies *mycoides* small colony. It induces significant economic losses and leads to a serious food security problem, negatively influencing peoples' livelihoods in affected countries. The disease has been reported in different parts of Ethiopia with prevalence ranging from 1.78 to 96%. However, there is not enough epidemiological information about CBPP in the northwestern part of the country, particularly in North Gondar Administrative Zone. This cross-sectional study, therefore, was conducted in four selected districts (Metema, Alefa, Quara, and Dembia) of North Gondar Administrative Zone to detect the incursion and estimate the seroprevalence of CBPP and to identify the potential predisposing factors associated with the spread and occurrence of CBPP in the area. A total of 751 serum samples were collected from 41 herds (villages) having no history of CBPP vaccination. Information like age, sex, breed, herd size, and management were collected during blood sample collection. Data related to agro-climatic zone, trade trekking route from or through CBPP endemic/epidemic zone, transhumance grazing route, vehicle route, and bordering with CBPP epidemic/endemic zone were obtained from district agricultural offices. The serum was screened for CBPP using competitive enzyme-linked immunosorbent assay (c-ELISA) test. The animal level and herd level apparent seroprevalences were 12.92% (95% CI: 10.70–15.52) and 65.85% (95% CI: 49.57–79.10), respectively. The true animal level and herd level prevalences were estimated at 20.13% (95% CI: 16.64–24.21) and 65.22% (95% CI: 48.64–78.72%), respectively. At the animal level, trekking route from or through CBPP endemic/epidemic zone to the study area [OR = 4.77 (95% CI: 1.92–11.84) compared to without trekking route] was identified as the most important risk factor for CBPP spread and seropositivity. In general, epidemiological evidence of the present study indicates that CBPP is a prevalent disease, and animal trekking is an important risk factor for spread of the disease in the study area. This needs due attention from the government and other concerned bodies for its prevention and control to mitigate its economic impact.

## Introduction

Contagious bovine pleuropneumonia (CBPP) is an infectious and highly contagious respiratory disease of cattle and water buffalo, which is caused by the *Mycoplasma mycoides* subspecies *mycoides* small colony (*MmmSC)*. It is one of the most important diseases in many sub-Saharan African countries (1–5). CBPP is a notifiable disease in almost all African countries (1). In 2006, 20 African countries reported outbreaks of the disease with the highest number of cases from Ethiopia, Cameroon, and Angola (3). In affected countries where the disease is prevalent, it has serious consequences on food security and peoples' livelihoods (4).

Contagious bovine pleuropneumonia transmission occurs through direct and repeated contacts between infected and susceptible animals. Inhalation of infective droplets is the main route of infection (2). Under natural conditions, the main source of infection is the excretion of infective droplets by the infected coughing animal. Nasal discharge, fetal fluids, and urine of sick animals can serve as sources of infection (6). Crowding of animals around watering points or at night in a paddock or pen facilitates the transmission of the disease (7). Factors such as crowding, genetic constitution, age, intercurrent infections, inclement climatic conditions, agro-ecological zone and stress from handling, experimentation, and transportation are important determinants for the occurrence of CBPP (4, 8, 9). The management system heavily influences the occurrence and incidence of CBPP (2). Cattle movements are responsible for the spread of CBPP from one herd, region, or country to another. Hence, the type of husbandry employed plays an important role in the epidemiology of the disease (10).

In affected cattle, the disease can be manifested in hyper acute, acute, subacute, or chronic forms. Fever, depression, anorexia, agalactia, and respiratory signs such as cough, nasal discharges, dyspnea, and polypnoea characterize the disease. The disease is also characterized by lesions like unilateral pneumonia associated with pleurisy, consolidation and marbling of lung, and pleural adhesion. In chronic stage of the disease, lungs may develop encapsulated lesions called sequestra. Animals with sequestrated lesions may be long-term carriers and are potential shedders of the organism (1, 2, 5, 11). Older animals are more affected by the disease than young animals. Calves rarely show pneumonic diseases and as a result they play a minimal role in the spread of the disease (1).

CBPP outbreaks tend to be more common in housed animals and in those animals transit in-group. In naive animals, the morbidity and mortality of CBPP approaches 100 and 50%, respectively, and about 25% of the infected cattle became recovered carriers (2, 12). Animals recovered from CBPP are resistant to further infection (1).

Contagious bovine pleuropneumonia is considered to be a disease of economic importance because of its high morbidity and mortality rate, costs related to quarantine, increased cost of controlling the disease, delayed marketing, reduced draft power, reduced fertility, loss of market due to trade bans and inhibition of sustained investment in livestock production (2, 13). The financial and economic loss caused by the disease in Africa is significant. Masiga et al. (14) reported that the continent had lost approximately 2 billion US$ per year due to death of animals from the disease.

Diagnosis of CBPP is done based on clinical signs, isolation of the etiological agents, polymerase chain reaction (PCR), serological tests, and necropsy findings (2, 5). From serological tests, the enzyme-linked immunosorbent assays (ELISAs) and the complement fixation test (CFT) are recommended for screening and eradication programmes of CBPP (5). CFT is more sensitive in detecting sick animals with acute lesions while c-ELISA is more sensitive in cattle with lesions in the chronic stage (5). All stages of CBPP infection could not be detected by a single serological test; therefore, use of more than one test is advisable (15).

In Ethiopia, after Rinderpest has been brought under control, CBPP is considered to be among the most important cattle diseases that hinders livestock development (16). Studies undertaken on CBPP so far in Ethiopia revealed the existence of the disease in different parts of the country with prevalence varying from 1.78% in southern and Eastern lowlands (17) to 96% in Western Gojjam (18). The disease is causing high economic losses on the agricultural sector and the national economy. It accounts for losses of over 205.6 million Ethiopian birr per year (19). Available data on the distribution of the disease in Ethiopia show the northwestern part of the country, especially the western part of the North Gondar zone (where livestock production is very important), as free or status unknown for CBPP. This area is adjacent to the endemic zone of west Gojjam and Awi, epidemic zone of Benshangul-Gumuz, and unknown status of South Gondar zone where the status of the disease is unknown too (20). As there is no animal movement restriction in place, it is highly likely that the disease could have been spread to this area.

Generally, although the disease brings high economic loss in the livestock industry, there is not enough information and research works regarding predisposing factors and prevalence of CBPP in the northwestern part of the country particularly in the western part of the North Gondar administrative zone. Hence, this study was initiated to detect the incursion and estimate the seroprevalence of CBPP in the western part of the North Gondar administrative zone and to identify the potential predisposing factors associated with the spread and occurrence of CBPP.

## Materials and Methods

### Study Area

**Figure 1:**
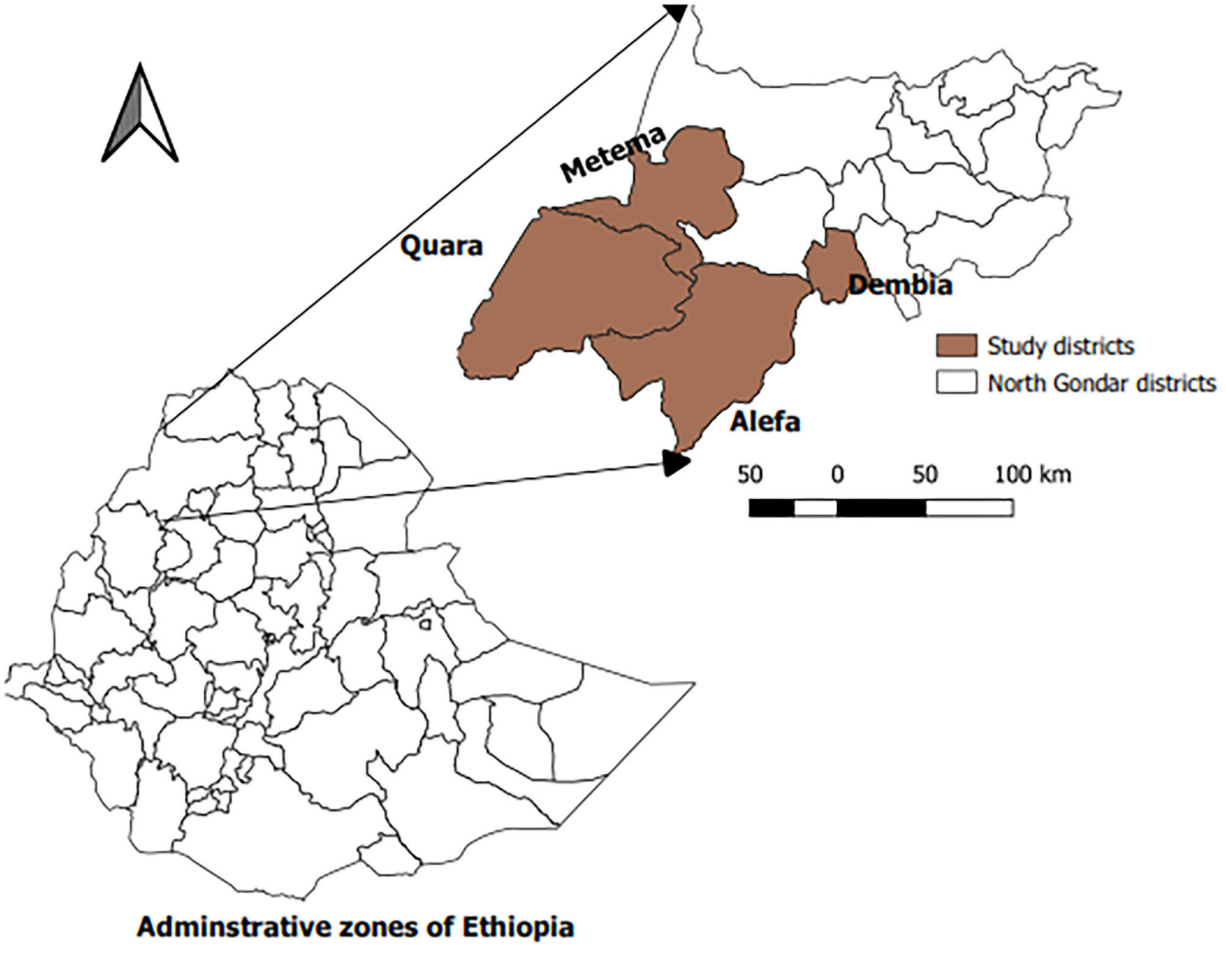
Map of Ethiopia showing the study districts in North Gondar zone.

The study was conducted in four selected districts (Metema, Alefa, Quara, and Dembia) of North Gondar administrative zone (Figure 1). North Gondar zone, located in the north west of the region, is one of the 11 administrative zones in the Amhara National Regional State. It is divided in to 22 administrative districts. Geographically, the area lies between 12.3–13.8°N latitude and 35.35–38.50°E longitude. The zone has a cattle population of 3,525,578, which is kept dominantly under the traditional smallholder farming system (21).

### Study Population and Design

The source population comprises all cattle in the study districts with no history of recent vaccination against CBPP.

A cross sectional study with a four-stage sampling technique was carried out in four districts from October 2011 to May 2012. Cattle were selected by multistage cluster sampling in which district was the primary sampling unit, kebele the secondary sampling unit, herd (village) tertiary sampling unit and individual animals the fourth sampling unit. Blood sample for serology was collected from each animal using plain vacutainer tubes and information like age, sex, breed, herd size, and management was collected at the time of sampling. Data related to agro-climatic zone, trade trekking route from or through CBPP endemic/epidemic zone, transhumance grazing route, vehicle route, and bordering with CBPP epidemic/endemic zone were obtained from district agriculture offices. The serum was screened for CBPP using c-ELISA test at National Animal Health Diagnostic and Investigation Center (NAHDIC), Ethiopia.

### Sample Size Determination and Sampling Procedures

**Figure 2:**
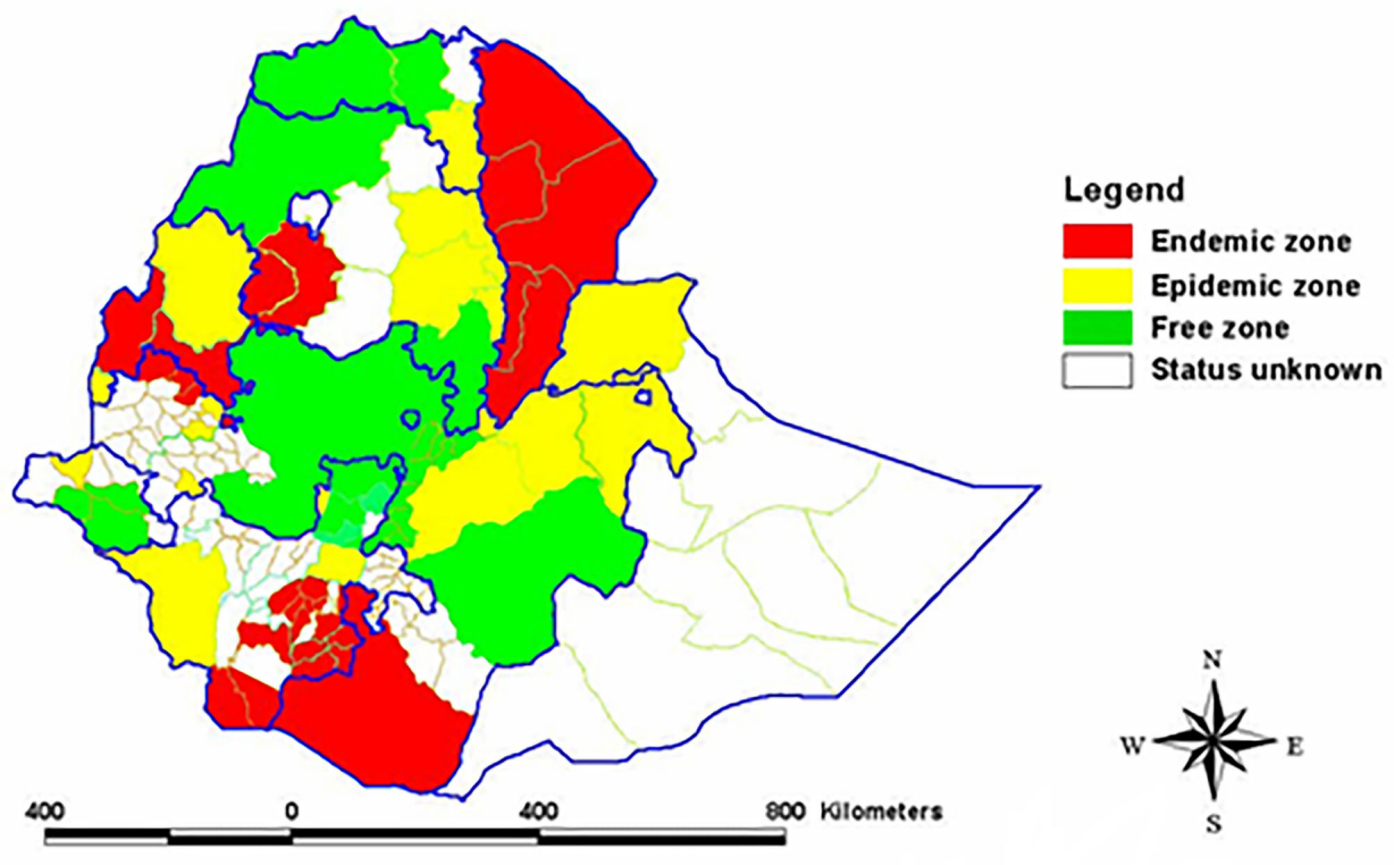
Map showing the different CBPP zones of Ethiopia. Source: (20).

The sampling technique employed was multistage cluster sampling in which the first selection was done purposely and selection from the next stratum was random. Accordingly, selection at district level was purposive. Thus, the study districts: Metema, Alefa, Quara, and Dembia were selected for the study based on the presence of cattle trading route crossing through them and their proximity to CBPP endemic or epidemic zone (Figure 2). Kebele within district, herd within kebele, and individual animal within herd were selected randomly. Herd here refers to a group of animals living in a village and share common grazing and watering points. The sample size (n) was determined based on the formula provided by Bennett et al. (22).

(1) $$\begin{array}{r} n=gc=\frac{P\left(100-P\right)D}{SE^{2}} \end{array}$$

Where p is the prevalence as percentage, D is the design effect, SE is the precision, c is the number of clusters (herds) sampled and g is the average number of animals sampled per herd (cluster).

Formula 2 gives the design effect:

(2) $$\begin{array}{r} D=1+\left(g-1\right)ICC \end{array}$$

Otte and Gumm (23) reported that the estimate of ICC (intracluster correlation coefficient) for most infectious diseases rarely exceed 0.20. Thus, assuming a value of ICC of 0.20, the design effect (D) of a cluster-sample survey with a sample of 24 animals per cluster is 5.6 (Formula 2). Sampling 24 animals per cluster (herd) with an expected disease prevalence of 9.1% (24) and a desired precision of 2.5% gave 31 clusters and thus a total sample size of around 744 animals. The lists of kebeles, herds and animals were obtained from district agricultural offices and the respective selected kebele development agents. As all cattle in a village (herd) shared common grazing and watering points, they were considered as one population (cluster).

### Sample Collection and Test Procedure

#### Blood Sample Collection

Blood sample collection for serological examination was performed after properly restraining the animal and disinfecting the skin over the jugular vein with 70% alcohol. About 6 ml of blood was withdrawn from each selected animal using a plain vacutainer tube and the blood was allowed to clot at room temperature. Then the serum sample was separated from the clotted blood by centrifugation and stored at −20°C until testing. Each serum sample was labeled legibly.

#### Serological Test Procedure

The c-ELISA test was used for the detection of anti-CBPP antibodies as described by the OIE (5). C-ELISA material for CBPP testing was provided in sealed bags as a ready-made kit that contains all the necessary reagents, including precoated plates. The serological analysis was performed following the manufacturer's protocol and recommendation. Each serum sample was mixed and diluted with specific monoclonal antibody (Mab 117/5) in a dilution plate and was incubated at 37°C for 1 h. After washing, anti-mouse immunoglobulin G serum conjugated to horseradish peroxidase was added and incubated again for 30 min. Following another series of washes, substrate was added and incubated for 30 min at +37°C. Then stop solution was added and shacked gently until the colored solution was homogenized. Finally, immediately following the addition of the stop solution the optical densities were read at 450 nm (OD 450) after blanking the photometer (ELISA reader) on air.

The percentage of inhibition (PI) for each serum was calculated using formula 3.

(3) $$\begin{array}{r} Percentageofinhibition=\frac{100\;\left(\left(OD*CM\right)-\left(OD*Test\right)\right)}{\left(\left(OD*CM\right)-\left(OD*Cc\right)\right)} \end{array}$$

Where OD = optical density, Cm = monoclonal control, Test = test serum, and Cc = conjugate control. Sera samples with percentage of inhibition equal to or <50% were considered positive for CBPP antibody.

### Methods of Data Management and Statistical Analysis

The collected data and laboratory findings were entered and stored in MS-excel. Before being subjected to statistical analysis, the data were thoroughly screened for errors and properly coded. Stata 14 software package was used to perform the statistical analysis.

Descriptive statistics such as tables were used to summarize and present the data collected. CBPP prevalence was calculated as percentage by dividing the number of samples positive for *MmmSC* antibody to the total number of serum samples tested. The herd level and animal level true prevalences were calculated by adjusting the corresponding apparent seroprevalence (AP) for specificity (Sp) and sensitivity (Se) of c-ELISA or herd specificity (HSp) and herd sensitivity (HSe) using formula 4 (25). The specificity and sensitivity of c-ELISA are 99.9 and 63.8%, respectively (5).

(4) $$\begin{array}{r} \text{True prevalence }=\;\frac{\text{Apparent prevalence}+\text{Specificity of the test}-1}{\text{Sensitivity of the test}+\text{Specificity of the test}-1} \end{array}$$

The herd level Sp and Se were derived based on formula 5 and 6 (26).

(5) $$\begin{array}{r} HSp=Sp^{\text{n}} \end{array}$$

(6) $$\begin{array}{r} HSe=1-{\left(1-Ap\right)}^{\text{n}} \end{array}$$

Where n is the average sample size per herd and the herd is considered positive if at least one of the animals within it was tested positive.

Multilevel mixed effect logistic regression model taking herd as a random effect was employed to assess the existence of association between serological results (dependent variable) and independent variables (age, sex, breed, herd size, agro-climatic zone, trade trekking route from or through CBPP endemic/epidemic zone, transhumance grazing route, vehicle trade route, bordering with CBPP epidemic/endemic zone, and distance from epidemic and endemic zone). Following the univariable analysis, those variable with *P* <0.25 and not collinear with each other were fitted in a multivariable model. The final model was obtained by a backward stepwise elimination procedure while checking for confounding. Confounding was considered present if there is at least a 25% change in the coefficients of any of the remaining variables after removing a non-significant value (*p* >0.05) from the model.

## Results

### Seroprevalence

Contagious bovine pleuropneumonia seropositive animals were detected in all study districts. Of all animals sampled, 97 [12.92% (95% CI: 10.70–15.52)] were tested positive for CBPP antibody while 27 herds [65.85% (95% CI: 49.57–79.10)] had at least one positive animal for CBPP antibody. The highest animal level and herd level apparent prevalences were recorded in Quara district whereas the lowest in Dembia district (Table 1). Both the animal level and herd level prevalences varied significantly among the districts.

**Table 1:** Animal and herd level CBPP seroprevalence by district in North Gondar zone, Ethiopia.

**District**	**Animal level**			**Herd level**		
	**No. of animals sampled**	**No. of positive animals**	**Apparent prevalence (95% CI)**	**No. of herds sampled**	**No. of positive Herds^a^**	**Apparent prevalence (95% CI)**
Quara	251	44	17.53 (13.30–22.76)	11	11	100
Metema	102	13	12.75 (7.52–20.79)	7	5	71.43 (28.70–93.95)
Dembia	199	8	4.02 (2.02–7.85)	11	3	27.27 (8.20–61.16)
Alefa	199	32	16.08 (11.59–21.88)	12	8	66.67 (35.44–87.93)
**Overall**	751	97	12.92 (10.70–15.52)	41	27	65.85 (49.57–79.10)

a *A herd is positive if it has at least one animal tested positive*.

*Difference between districts*:

*Animal level: Chi-square = 20.53 (3 df), P < 0.001*.

*Herd level: Fisher's exact = P = 0.002*.

The true overall animal level prevalence was estimated at 20.13% (95% CI: 16.64–24.21) by considering the 63.8% sensitivity and 99.9% specificity of c-ELISA. The true overall herd level seroprevalence was also estimated as 65.22% (95% CI: 48.64–78.72%) by deriving herd sensitivity (HSe = 100%) and herd specificity (HSp = 98.2%) using the animal level Se (63.8%), Sp (99.9%), and average sample size per herd (*n* = 18).

### Risk Factors for CBPP Serostatus

The univariable mixed effect logistic regression results are presented in Table 2. Age, trekking route from or through CBPP endemic/epidemic zone, bordered with epidemic/endemic zone, and distance from endemic/epidemic zone were selected for the multivariable model (*p* <0.25).

**Table 2:** Univariable analyses of potential risk factors for CBPP at animal level (*n* = 751) using mixed effect logistic regression model including herd as random effect.

**Risk factors**	**Category**	**Number of animals tested**	**Number (%) of seropositive**	**Odd ratio**	**Overall *P*-value**
Agroclimate	Lowland	353	57 (16.15)		0.301
	Midhihgland	398	40 (10.05)	0.54	
Herd size	Small	104	11 (10.58)		0.5867
	Medium	288	32 (11.11)	1.72	
	Large	359	54 (15.04)	1.19	
Breed	Local Zebu	738	97 (13.14)		
	HF cross	13	0 (0.00)		
Sex	Female	471	62 (13.16)		0.936
	Male	280	35 (12.50)	0.98	
Age group	0.5 ≤ 1 year	63	4 (6.35)		0.1322
	1–3 years	200	20 (10.00)	1.58	
	≥3 years	488	73 (14.96)	2.37	
Route for grazing	No	199	32 (16.08)		0.394
	Yes	552	65 (11.78)	0.56	
Trekking from or through CBPP endemic/epid. zone	No	199	8 (4.02)		0.001
	Yes	552	89 (16.12)	4.77	
Vehicle route	No	450	76 (16.89)		0.945
	Yes	301	21 (6.98)	1.06	
Border	No	301	21 (6.98)		0.0133
	With endemic	199	32 (16.08)	2.85	
	With epidemic	251	44 (17.53)	3.13	
Distance	Adjacent^*^	450	76 (16.89)		0.0023
	Intermediate^**^	199	8 (4.02)	0.19	
	Far^***^	102	13 (12.75)	0.67	

* *adjacent if the distance is <50 Km*,

** Intermediate if the distance is from 50 to 100 Km, and

*** *Far if the distance is >100 Km*.

The final multivariable mixed effect logistic model identified trekking route from or through CBPP endemic/epidemic zone to the study area, OR = 4.77 (95% CI: 1.92–11.84), as the most important risk factor in relation to CBPP spread and seroprevalence. The inter-herd variance was estimated as 0.370 resulting in an ICC of 0.101 (95% CI: 0.030–0.288).

## Discussion

Seroepidemiological study is one of the means to establish presence and prevalence of transboundary animal diseases such as CBPP in an area. In Ethiopia, the serological survey carried out by National Veterinary Institute (NVI) from 1995 to 1997 has classified the country into endemic, epidemic, and free zones for CBPP (20, 27). Based on this classification, North Gondar administrative zone was categorized under CBPP free zone. However, in the current study, CBPP antibody positive cattle were detected in the four studied districts. Finding CBPP antibody positive cattle in the zone, which was considered free, is not surprising as there is free animal movement into and out of the study districts from different parts of the country for trading and grazing purposes and such uncontrolled animal movements facilitate the spread of CBPP (28). Besides, some of these districts share borders with epidemic or endemic zones and countries. Whatever the case may be, this study results refute the view that northwestern part of the country (particularly North Gondar administrative zone) is free from CBPP (20, 29).

The present study had shown that CBPP is prevalent in North Gondar administrative zone because about 66% of the herds (villages) and 13% of the animals tested were found positive for CBPP. These observed prevalences seem to be high for an area where CBPP has never been reported and has been considered free from the disease. The 12.92% animal level apparent seroprevalence of CBPP estimated in this study is comparable to the 12% prevalence reported in Borana (30) and 11.6% in Shinile (31). However, it is low when compared to the 96% reported in Western Gojjam, 75% in west Wellega, 74% in Borana and 59% in Southern Tigray (18), 48% in West Wellega (32), 46% in Konso, 32% Derashe, and 29% in South Omo (33). CBPP prevalence lower than the current one has also been reported in Arbaminch and Shashemene (34), Jijiga (31), Guji (30), and at export quarantine centers in and around Adama (35). The variation in these observed prevalence levels could be accounted to the differences in the types of tests used, year of study, agro-ecological zones, herd size, breed susceptibility, animal management and production system, seasonal herding patterns, and contact patterns. In this study, we used only c-ELISA tests to categorize cattle as CBPP seropositive and negative. This test is more sensitive in detecting cattle at the chronic stage, but it is less sensitive in detecting animals at the early stage of CBPP infection (15). This property of the test might have resulted in underestimation of the seroprevalence of the disease reported in the study area and this can be taken as a weakness of the study. Thus, the use of an additional test should be considered in future studies.

In this study, a great spatial variability across the study districts was observed. Indeed, a moderate animal level and high herd level prevalences were observed in Quara, Metema, and Alefa districts. This perhaps is due to the fact that these districts have more trekking routes and share boarder with known CBPP endemic and epidemic zones, regions, and neighboring country Sudan. However, in the Dembia district where there is no known trekking route and no shared border with CBPP epidemic or endemic zones, regions, and countries, the seroprevalence of CBPP was moderate at herd level and low at animal level.

Among the studied factors, the presence of trekking route from or through CBPP endemic/epidemic zone to the study area was associated with CBPP seroprevalence. Seroprevalence of CBPP was almost five times (OR = 4.77) higher in districts which had trekking route from or through CBPP endemic/epidemic zone compared to districts without trekking route. Because of difference in price of cattle, traders trek animals from long distance across the districts and to Sudan in search of better price for their cattle. Cattle trekked through this study area often stop at several points along the way, thus exposing animals reared along these trade routes to CBPP (36). This finding is consistent with the result of Alhaji and Babalobi (9), who described that movements of cattle across states contributed to the occurrence of CBPP. Similarly, Provost et al. (6) and Masiga et al. (37) reported that cattle movements due to nomadism, transhumance, and trekking are responsible for the maintenance and spread of the disease within and across country borders. However, transporting animals by vehicle into and crossing through the study districts did not show significant association with CBPP seropositivity. As CBPP transmission usually occurs by direct or close contact (over distances <100 m) between infected and susceptible cattle (38), vehicle transportation probably did not allow close contact sufficient for the disease transmission to animals along the road.

In this study, CBPP was detected in previously uninfected areas of North Gondar administrative zone. The underlying factor for the spread of the disease to these previously free areas might be uncontrolled or illegal movement of cattle from known infected cattle populations as indicated by other researchers (4). Failure to enforce disease control measures, absence of cattle identification and tracking system, weak surveillance system, and traditional livestock husbandry might have contributed for the incursion of the disease into the study area. CBPP outbreaks usually begin following effective contact of an infected animal with a naive herd as the result of uncontrolled animal movement (39). Moreover, absence of vaccination and quarantine often lead to the risk of introduction of CBPP into an area (29).

The current study area is located in the northwestern region of Ethiopia where one of the disease-free zones in the country is proposed for export (29). However, the present study showed high prevalence of CBPP in the area. This urges a need for proper attention to control the disease. In relation to this regular annual vaccination and movement regulation can be considered appropriate prevention and control measures for the area. Animal movement regulation should be enforced in the area by improving animal identification and tracking scheme, updating stock routes, and defining entry requirements of animals into disease free zone. Vaccination is also a valuable tool in CBPP prevention and control. It is even more important in places like the current study area where movement regulation is relatively less feasible and needs much more effort.

In conclusion, the results of the present study indicate that CBPP is prevalent in North Gondar zone. Individual and herd level prevalences were higher in the districts bordering epidemic or endemic zones and in districts adjacent to Sudan. The greatest CBPP risk was observed in districts, which had trekking route from or through CBPP endemic/epidemic zones. Generally, the detection of many CBPP seropositive cattle in the area, which has been considered free of CBPP, indicate the continuing geographical expansion of the disease in the country. Therefore, further organized study covering all parts the country is suggested to motivate and shift the government attention toward the control and prevention of CBPP.

## Data Availability Statement

The raw data supporting the conclusions of this article will be made available by the authors, without undue reservation.

## Ethics Statement

Ethical review and approval was not required for the study on animals in accordance with the local legislation and institutional requirements. Written informed consent was obtained from the owners for the participation of their animals in this study.

## Author Contributions

WM, WJ, and SM conceived, designed the study, and drafted the manuscript. WM and SM undertook the data collection. GT and GA carried out the serological testing. WM and WJ analyzed and interpreted the data. All authors read, revised, and approved the final version of the manuscript for publication.

## Conflict of Interest

The authors declare that the research was conducted in the absence of any commercial or financial relationships that could be construed as a potential conflict of interest.
